# Repurposing of abemaciclib to overcome C797S-mediated osimertinib resistance in EGFR-mutant lung adenocarcinoma

**DOI:** 10.1186/s12929-026-01297-0

**Published:** 2026-11-12

**Authors:** Hsin-Jung Li, Chao-Chi Ho, Ci-Ling Chen, Chi-Yuan Chen, Ting-Jen Rachel Cheng, Ya-Hsuan Chang, Yu-Han Huang, Ming-Shiu Lin, Zi-Ling Lin, Shuenn-Chen Yang, Shih-Chien Huang, Yu-Ya Hung, Kuo-Yen Huang, Pan-Chyr Yang

**Affiliations:** 1 NTU YongLin Institute of Health, National Taiwan University, Taipei, 106 Taiwan; 2 Department of Internal Medicine, National Taiwan University Hospital, Taipei, 100 Taiwan; 3 Program for Precision Health and Intelligent Medicine, Graduate School of Advanced Technology, National Taiwan University, Taipei, 106 Taiwan; 4 Graduate Institute of Health Industry Technology and Research Center for Food and Cosmetic Safety, Chang Gung University of Science and Technology, Taoyuan, 613 Taiwan; 5 BioBank, Chang Gung Memorial Hospital, Taoyuan, Taiwan; 6 Genomics Research Center, Academia Sinica, Taipei, 115 Taiwan; 7 Institute of Molecular and Genomic Medicine, National Health Research Institutes, Zhunan, Miaoli 350 Taiwan; 8 Institute of Biomedical Sciences, Academia Sinica, Taipei, 115 Taiwan; 9 Department of Clinical Laboratory Sciences and Medical Biotechnology, College of Medicine, National Taiwan University, Taipei, 100 Taiwan; 10 NTU Advanced Biomedical Research Center, National Taiwan University, Taipei, 106 Taiwan

**Keywords:** EGFR C797S mutation, Osimertinib resistance, Abemaciclib, Drug repurposing, Kinase inhibitor selectivity, PDX models

## Abstract

**Background:**

Acquired resistance to osimertinib mediated by the EGFR C797S mutation remains a major therapeutic challenge in EGFR-mutant non–small cell lung cancer (NSCLC), with limited effective treatment options. This study explored whether abemaciclib, an FDA-approved CDK4/6 inhibitor, can overcome C797S-mediated osimertinib resistance in EGFR-mutant NSCLC.

**Methods:**

A high-throughput screen of approximately 125,000 compounds was performed using Ba/F3 cells expressing EGFR triple mutations (L858R/T790M/C797S and Del19/T790M/C797S). The impact of abemaciclib on EGFR kinase activity was evaluated via *in vitro* kinase and Western blot assays. Apoptosis induction was assessed using flow cytometry. The activity of abemaciclib was evaluated using biochemical assays, cellular thermal shift assays (CETSA), molecular docking analyses, CRISPR-engineered isogenic models, xenografts, and patient-derived xenograft (PDX) models.

**Results:**

Abemaciclib was identified as a potent inhibitor of EGFR triple-mutant cell growth and exhibited preferential activity toward mutant EGFR-dependent models. IL-3 rescue assays demonstrated that its antiproliferative effects were dependent on suppression of oncogenic EGFR signaling. Abemaciclib inhibited mutant EGFR kinase activity, reduced EGFR phosphorylation, and induced apoptotic cell death in osimertinib-resistant models while exhibiting substantially weaker activity toward wild-type EGFR. CETSA and molecular docking analyses supported direct engagement of mutant EGFR by abemaciclib. Importantly, abemaciclib significantly suppressed tumor growth in CRISPR-engineered isogenic xenografts and PDX models harboring EGFR C797S-mediated osimertinib resistance.

**Conclusions:**

Abemaciclib selectively suppresses C797S-mutant EGFR signaling and demonstrates robust antitumor activity in preclinical models of osimertinib-resistant EGFR-mutant NSCLC. This breakthrough not only overcomes the existing therapeutic barrier but also demonstrates minimal interference with WT EGFR, offering a transformative and precise therapeutic strategy with significant translational and clinical potential. These findings support the therapeutic repurposing of abemaciclib as a potential treatment strategy for EGFR C797S-mediated resistance and provide a translational strategy for overcoming resistance-associated EGFR alterations using clinically available targeted agents.

**Supplementary Information:**

The online version contains supplementary material available at https://doi.org/10.1186/s12929-026-01297-0.

## Introduction

Non-small cell lung cancer (NSCLC) accounts for approximately 85% of all lung cancer cases and remains a leading cause of cancer-related deaths globally [1]. Aberrant EGFR signaling is observed in ~40% of Asian and ~15% of Caucasian patients with NSCLC [2]. EGFR activation induces kinase activity and trans-autophosphorylation of the C-terminal tails of two receptor monomers. These tails contain tyrosine residues that, upon phosphorylation, serve as docking sites for effector proteins and facilitate downstream signaling. The principal sites are Y974, Y992, Y1068, Y1086, Y1148, and Y1173, which recruit downstream signaling proteins [3]. In particular, phosphorylation at Y1068 and Y1086 regulates EGFR kinase activity and promotes cell proliferation and survival [4].

In adenocarcinoma, common EGFR mutations include exon 19 in-frame deletions and the exon 21 L858R point mutation, which cause ligand-independent receptor activation [5–8]. These gain-of-function mutations sensitize tumors to first- and second-generation EGFR tyrosine kinase inhibitors (TKIs), including gefitinib, erlotinib, and afatinib [9–12]. However, resistance often develops, most commonly through the exon 20 T790M mutation (~50% of cases), which reduces TKI binding affinity [12–14]. Third-generation EGFR-TKIs, including EGF816, rociletinib, and osimertinib, irreversibly bind cysteine 797 (C797) to overcome this resistance [15]. Nevertheless, resistance may emerge through the C797S mutation (~15% of patients after osimertinib treatment), which can be detected in plasma DNA [15–18]. The C797S mutation is characterized by the substitution of cysteine with serine at codon 797 within the ATP-binding site of the EGFR tyrosine kinase domain [19]. Osimertinib covalently binds to EGFR at C797, while its pyrimidine core forms two stable hydrogen bonds with M793 [20]. The C797S substitution disrupts this interaction and markedly diminishes osimertinib activity, resulting in a 100- to 1,000-fold reduction in its ability to inhibit cell proliferation and EGFR phosphorylation [20]. This suggests that these point mutations can cause steric interference, resulting in reduced affinity of these compounds to the EGFR kinase domain [21]. Additional mutations such as L844V and L718Q, when expressed in cis with activating EGFR mutations, also confer resistance in preclinical models [15, 22]. These findings underscore the growing need for new strategies to overcome resistance in osimertinib-resistant patients.

Recent research has highlighted the potential of the allosteric EGFR small-molecule inhibitor EAI045, in combination with cetuximab, to suppress tumor growth in mouse models of lung cancer driven by EGFR-LTC (L858R/T790M/C797S). However, this combination is less effective in mice bearing EGFR-DTC (Del19/T790M/C797S) tumors [13]. Additionally, Uchibori et al [23] demonstrated that combining the ALK inhibitor brigatinib with EGFR antibodies such as cetuximab or panitumumab effectively suppressed tumor growth in a mouse xenograft model overexpressing EGFR-DTC. Both of these small molecules necessitate coadministration with cetuximab to suppress tumor growth. Given the increasing exploration of inhibitors capable of overcoming the effect of EGFR-DTC mutation on NSCLC [24, 25], there is an urgent need to address osimertinib-resistant EGFR-triple-mutant NSCLC in clinical settings.

Drug repurposing has emerged as an attractive strategy for accelerating the development of targeted therapies because clinically approved agents possess established pharmacologic and safety profiles that may facilitate rapid clinical translation. In the present study, we performed a high-throughput compound screen to identify therapeutically relevant inhibitors capable of suppressing EGFR C797S-mediated resistance. Through this approach, we identified abemaciclib, an FDA-approved CDK4/6 inhibitor [26, 27], as a potent suppressor of osimertinib-resistant EGFR signaling. Using biochemical assays, CRISPR-engineered isogenic models, xenografts, and patient-derived xenograft (PDX) models, we demonstrate that abemaciclib selectively suppresses mutant EGFR signaling and exhibits robust antitumor activity in multiple clinically relevant models harboring EGFR triple mutations. These findings support therapeutic repurposing of abemaciclib as a potential treatment strategy for osimertinib-resistant EGFR-mutant NSCLC.

## Methods

### Cell lines

BEAS-2B (immortalized human bronchial epithelial cell line) (EGFR wild-type), H460 (EGFR wild-type), Hop92 (EGFR wild-type), H1975 (EGFR mutant (T790M/C797S)), HCC827 (EGFR mutant (Exon 19 deletion)), and the virus packaging cell line HEK293 were purchased from the American Type Culture Collection (ATCC) (Manassas, VA, USA). H1975-35, H1975-12, and HCC827-2 cells were established using CRISPR/Cas9 to edit the codon of EGFR. CLH206 and VGHT11 cells, which were isolated from patient-derived lung tumors, were subcutaneously implanted into mice expressing triple-mutant EGFR (DTC). The murine pro-B-cell line Ba/F3 was obtained from the RIKEN BioResource Center (Tsukuba, Japan). We maintained parental Ba/F3 cells in the presence of murine interleukin-3 (IL-3) under sterile conditions. A549 and HEK293 cells were cultured in Dulbecco's modified Eagle’s medium (DMEM) supplemented with 10% fetal bovine serum (FBS) (Life Technologies); H1975 and HCC827 isogenic line cells were cultured in RPMI 1640 medium supplemented with 10% FBS; and CLH206 and VGHT11 cells were cultured in RPMI 1640 medium supplemented with 10% heat-inactivated FBS (HyClone, GE Healthcare) at 37 °C in a humidified atmosphere containing 5% CO_2_ and 20% O_2_. All the cell lines tested negative for mycoplasma (Mycoplasma PCR Detection Kit (abm)) throughout the study.

### High-throughput screening (HTS)

Compound screening was performed at the Ultra High-Throughput Drug Screening (uHTS) Facility established at the Genomics Research Center, Academia Sinica (GRC), Taipei, Taiwan (https://uhts.genomics.sinica.edu.tw/web/index.php). For preliminary screening (125311 compounds), EGFR-TKI-resistant Ba/F3 cells expressing triple-mutant EGFR were dispensed into the wells of a 1536-well plate at a concentration of 500 cells per well. Only 2633 compounds inhibited cell growth at 1 μM, and we further determined the fold change in IC50 values in the presence or absence of 3 ng/ml IL-3 in the culture media. Among the top 20 compounds identified in the screening, only abemaciclib was associated with the best left-shift in the IC50 value in cell proliferation assays. We selected abemaciclib for the following experiments.

### Knock-in of EGFR C797S mutation by CRISPR/Cas9

Using CRISPR/Cas9, isogenic H1975 (H1975-35 and H1975-12) harboring the EGFR L858R/T790M/C797S and HCC827 (HCC827-2) cell lines harboring the EGFR Del19/T790M/C797S mutations were generated respectively. The H1975-35 and H1975-12 cell lines were engineered to knock in C797S, whereas HCC827-2 cells were engineered to knock in T790M and C797S. A guide sequence (5′-CCT GGA CTA TGT CCG GGA AC-3′) specific to human EGFR exon 20 was used. We designed a single-stranded DNA donor template (5′-CCT CCA CCG TGC AAC TCA TCA TGC AGC TCA TGC CCT TCG GCA GTT TAC TGG ACT ATG TCC GGG AAC ACA AAG ACA ATA TTG GCT CCC AGT ACC TGC TCA ACT CA-3′) for precise genome editing. H1975 cells were cotransfected with CRISPR-EGFR sgRNA and a DNA donor template with Lipofectamine 2000 (Life Technology), after which successful cells were selected using culture medium supplemented with puromycin and osimertinib. Afterward, the surviving single cells were selected using flow cytometry sorting, and C797S-EGFR-knock-in into H1975 cells was validated by Sanger sequencing. The ability to overcome osimertinib resistance was confirmed via cell proliferation assays and western blotting.

### Cell viability assay

To determine cell viability, we utilized the MTS cell proliferation assay. In short, 4 × 10^3^ cells/well were placed in 96-well plates containing complete culture media before abemaciclib treatment. Following a 24-hour incubation period, the cells were treated with different concentrations of abemaciclib in complete culture media at 37 °C for an additional 72 hours. After treatment, 20 μl of MTS stock solution was added to each well and incubated for 1 hour. Absorption was then measured at 490 nm using a spectrophotometer (Molecular Devices). The 50% inhibitory concentration (IC50) value was determined as the drug concentration that resulted in a 50% reduction in absorbance after 72 hours of continuous drug exposure.

### Flow cytometric DNA content analysis and apoptosis assay

In brief, 4 × 10^3^ cells/well were placed in 6-well plates containing complete culture media overnight. After 24 hours, the cells were treated with different concentrations of abemaciclib or 0.1% DMSO as a vehicle control for the specified durations and then washed twice with cold PBS. Both the adherent and round cells were collected, fixed in 70% ethanol, and stained with 40 μg/ml of propidium iodide (PI) containing 20 μg/ml of RNase A for at least 30 minutes. The fluorescence intensity was measured using a FACSCalibur flow cytometer (BD Biosciences), and the subdiploid DNA content was analyzed using the CellQuest program (BD Biosciences). The caspase inhibitor Z-VAD-FMK (R&D Systems) was reconstituted according to the manufacturer's instructions and used at a final concentration of 10 μM. A total of 4 × 10^3^ cells/well were seeded in 6-well plates with complete culture media and left overnight. After 24 hours, the cells were treated with the caspase inhibitor Z-VAD-FMK or with DMSO as a vehicle control for 72 hours. The cells were subsequently washed twice with cold PBS. The cells were then harvested and tested for evidence of apoptosis using standard apoptosis assays.

### Antibodies, immunoprecipitation and immunoblotting

As previously described, immunoprecipitation (IP) and Western blotting were conducted [8]. In brief, the cells were lysed using IP lysis buffer, which consisted of 20 mM Tris (pH 7.5), 100 mM sodium chloride, 1% IGEPAL CA-630, 100 μM Na_3_VO_4_, 50 mM NaF, and 30 mM sodium pyrophosphate with complete protease inhibitor cocktail containing EDTA (Roche Diagnostics, Basel, Switzerland). Protein concentrations were measured using a Bio-Rad protein assay (Bio-Rad, Richmond, CA). For immunoprecipitation, 1 μg of primary antibodies and protein A beads (Sigma) were used to pull down the proteins, followed by three washes with lysis buffer. The protein samples were separated on 10% sodium dodecyl sulfate‒polyacrylamide gels, transferred onto poly-vinylidene difluoride membranes (Millipore, Billerica, MA, USA), and finally immunoblotted with primary antibodies. The following primer antibodies for immunoblotting were used at dilutions ranging from 1:1000 to 1:10000: anti-EGFR (1005), anti-AKT (H-136), anti-ERK (K-23), (Santa Cruz Biotechnology, Dallas, TX, USA), p-EGFR (p-1068), p-AKT (S473), p-ERK (T202/Y204), cleaved-PARP (Asp-214), cleaved-caspase 3 (Cell Signaling Technology, Danvers, MA, USA), anti-β-actin, and anti-Flag (M2; Sigma). Horseradish peroxidase-conjugated anti-mouse, anti-goat, and anti-rabbit secondary antibodies were used at a 1:5000 dilution. Protein signals were detected using Amersham Pharmacia, Piscataway, and NJ chemiluminescence reagents.

### Lentivirus production and transduction

We subcloned EGFR into the pLKO_AS2.neo lentivirus expression vector. Standard protocols were followed to produce the lentiviruses by the National RNAi Core Facility, Academia Sinica, Taipei, Taiwan. Briefly, Lipofectamine 2000 reagents (Life Technology) were used to cotransfect the indicated lentivirus plasmids and two helper plasmids, pCMVΔR8.91 and pMD. G, into HEK293 cells. Virus-containing medium was collected on days 1, 2, and 3 posttransfection. Cells were infected with the indicated lentiviruses in medium supplemented with 8 μg/ml polybrene. After 24 hours, the medium was replaced with fresh medium for another 24–48 hours. To generate stable infected cells, the cells were treated with 400 μg/ml of G418 to select antibiotic-resistant cells.

### In vitro EGFR kinase assay

The kinase assay was performed following the guidelines provided by the manufacturer of the ADP-Glo^™^ Kinase Assay (V9101; Promega). Briefly, 500 ng of recombinant EGFR protein (EGFR WT, Cat. 40187; EGFR L858R/T790M/C797S, Cat. 40351, BPS, Bioscience), 5 μg of substrate (poly (Glu-Tyr), P 0275, Sigma) and 250 picomole of ATP in EGRF kinase buffer (50 mM HEPES (pH 7.5), 10 mM MgCl_2_, 1 mM EGTA, 0.01% Brij-35) supplemented with vehicle or inhibitors were added in a total volume of 25 μl at room temperature for one hour. Afterward, 10 μl of kinase reaction mixture was added to 10 μl of ADP‐Glo™ Reagent for another 20 minutes. Afterward, 20 μl of Kinase Detection Reagent was used for the final 20 minutes, and the luminescence was recorded (integration time 0.5‐1 second) using a spectrophotometer (Molecular Devices).

### Human phosphokinase array

The detailed materials and methods for the human phosphokinase array (#ARY003B; R&D Systems) are comprehensively described in this paper [28].

### Cellular thermal shift assay (CETSA)

CETSA was performed to assess EGFR thermal stability upon drug engagement in intact H1975-35 cells. Cells were harvested, washed with PBS, and resuspended at 1 × 10^7^ cells/ml in PBS. Aliquots were divided into two groups and treated with DMSO (vehicle) or 1 μM abemaciclib for 1 h at 37°C. Treated suspensions (50 μl/tube) were heated across a thermal gradient (40–70 °C) for 3 min in a thermal cycler, cooled at room temperature for 3 min, and snap-chilled on ice for 3 min. An equal volume of 2× lysis buffer (100 mM Tris-HCl pH 7.5, 300 mM NaCl, 2% digitonin, 2× protease inhibitor cocktail) was added, followed by three freeze-thaw cycles (liquid N_2/37 °C water bath) for lysis. Lysates were centrifuged at 20,000 × *g* for 20–30 min at 4 °C; supernatants containing soluble proteins were recovered.

Supernatants were mixed with SDS loading buffer, incubated at 37 °C for 20 min (without boiling to avoid EGFR aggregation), resolved by SDS-PAGE, and immunoblotted with anti-EGFR antibody. Band intensities were quantified by densitometry, normalized to room temperature samples (set as 100%), and plotted as thermal melt curves.

### Molecular docking

Docking analyses of abemaciclib with EGFR L858R/T790M (PDB ID: 3IKA) and EGFR L858R/T790M/C797S (PDB ID: 6LUB) were conducted using BIOVIA Discovery Studio software (version 19.1.0.18287), as previously described [29].

### Xenograft mouse model

The tumor model was established in six-week-old male BALB/c nude mice (NARLabs, Taipei, Taiwan) subcutaneously inoculated in the right flank with 2 × 10^6^ H1975-LTC, H1975, H1975-35, 5 × 10^6^ HCC827-2, or 2.5× 10^6^ CLH206 cells in a total volume of 0.1 ml of sterile PBS (pH 7.4) on day 0. The mice were treated by oral gavage every day when the average tumor volume reached 150 mm^3^. The mice were randomized into four or six groups. The drugs were dissolved in oral gavage buffer (0.5% methylcellulose, 0.1% Tween 80) in sterile water at a volume of 200 μl per 20-gram body weight of mouse 5 days a week. Tumor sizes were measured five days per week using calipers, and the tumor volume was calculated according to the following formula: V = 0.5W^2^L (W: width represents small diameter; L: length represents large diameter) and statistical analysis of tumor volume was performed on the last day of treatment in different groups. Body weight was also measured five days per week. At the end of the study, the mice were sacrificed by CO_2_ asphyxiation, and the tumors were preserved in liquid nitrogen for EGFR protein detection. All experimental procedures were reviewed and approved by the Institutional Animal Care and Use Committee (IACUC), National Taiwan University. All animal experiments were conducted blindly, with injectors and data analysts unaware of the treatment groups.

### Statistical analysis

For the phosphorylated EGFR experiments, each experiment was performed with three independent replicates. Within each experiment, values for each treatment group were normalized to the corresponding control group, with the control value set to 100%. Each treatment group was then compared with the normalized control value of 100% using a two-tailed one-sample Student’s t-test. To account for multiple comparisons within each experiment, P values were adjusted using the Benjamini–Hochberg (BH) false discovery rate (FDR) procedure, and an adjusted P value < 0.05 was considered statistically significant. For all other experiments, comparisons between two groups were performed using two-sample t-tests. Equality of variances was first assessed using an F-test, followed by a Student’s t-test assuming equal variances or Welch’s t-test assuming unequal variances, as appropriate. Depending on the experimental design, comparisons were either restricted to prespecified pairs of interest or performed across all relevant pairwise combinations. When multiple comparisons were conducted within the same experiment, the resulting P values were adjusted using the Benjamini–Hochberg (BH) false discovery rate (FDR) procedure. An adjusted P value < 0.05 was considered statistically significant.

## Results

### High-throughput screening identifies abemaciclib as a selective suppressor of osimertinib-resistant mutant EGFR signaling

**Fig. 1:**
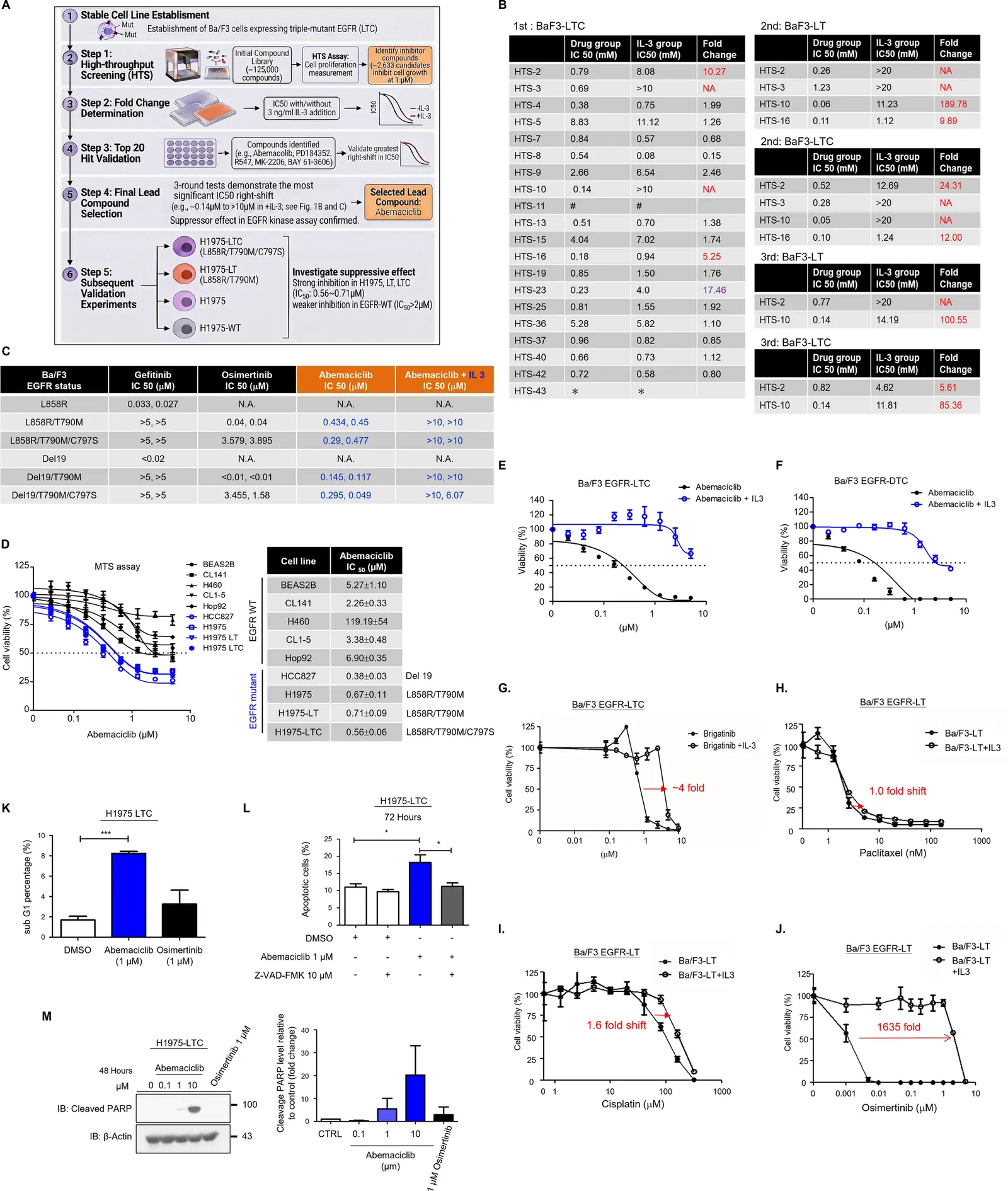
High-throughput screening identifies abemaciclib as a selective suppressor of osimertinib-resistant mutant EGFR signaling. **A** Schematic overview of the drug-screening protocol for the EGFR-LTC inhibitor abemaciclib. **B** A high-throughput screening assay of ~125,000 compounds was performed to measure cell proliferation of Ba/F3 cells expressing different mutant types of EGFR with or without IL-3. 1st: First-round drug selection; 2nd: second-round drug selection; 3rd: third-round drug selection. LT: L858R/T790M; LTC: L858R/T790M/C797S. #: The drug was ineffective; *: the drug did not dissolve; NA: not applicable. **C** A high-throughput screening assay of ~125,000 compounds was performed to measure cell proliferation in EGFR-TKI resistant Ba/F3 cells expressing mutant EGFR variants. **D** Cells were treated with abemaciclib at the indicated concentration for 72 hours. Cell viability was analyzed using the MTS assay, and the IC50 values were comparable (on the right). The error bars represent the means ± SDs; *n*=3. Ba/F3 EGFR-LTC (**E**) or Ba/F3 EGFR DTC cells (**F**) were treated with abemaciclib in the absence or presence of IL-3 at the indicated concentration for 72 hours, and cell viability was analyzed using the MTS assay. **G** EGFR-LTC cells were treated with brigatinib in the absence or presence of IL-3 at the indicated concentration for 72 hours, and cell viability was analyzed using the MTS assay. EGFR-LT cells were treated with paclitaxel (**H**), cisplatin (**I**), and osimertinib (**J**) in the absence or presence of IL-3 at the indicated concentration for 72 hours, and cell viability was analyzed using the MTS assay. **K** The proportions of H1975-LTC cells in the sub-G1 phase were determined after abemaciclib or osimertinib treatment. **L** Apoptosis assays were performed in H1975-LTC cells treated with the pancaspase inhibitor Z-VAD-FMK at the indicated concentration for 72 hours. **M** H1975-LTC cells were treated with the indicated concentration of abemaciclib for 48 hours, after which the cell lysates were harvested. Protein extracts were immunoblotted with the indicated antibodies. β-Actin served as a loading control in all the Western blotting panels

To identify small molecules that selectively target EGFR variants conferring resistance to targeted therapy, we established oncogene-dependent Ba/F3 cells stably expressing the triple-mutant EGFR (L858R/T790M/C797S) by lentiviral transduction (Supplementary Fig. 1A-C). We conducted a high-throughput phenotypic screen of approximately 125,000 compounds (Fig. 1A). Primary filtering yielded 2,633 compounds that inhibited cell proliferation at 1 μM, which were subsequently subjected to an IL-3 rescue assay to distinguish EGFR pathway-dependent effects. Among the candidate hits, abemaciclib emerged as the most prominent, exhibiting the largest rightward shift in IC_50_ upon IL-3 supplementation across independent screens (Fig. 1B, C). This shift, from 0.14 μM to >10 μM, indicates that its antiproliferative activity is highly dependent on oncogenic EGFR signaling. Consistent with this finding, abemaciclib directly suppressed EGFR kinase activity in triple-mutant systems (Supplementary Fig. 1D), supporting functional engagement of mutant EGFR. Notably, abemaciclib exhibited preferential activity in EGFR-mutant lung cancer models, including H1975, H1975-LT, and H1975-LTC cells, whereas its effects were substantially weaker in EGFR wild-type cells (IC_50_ > 2 μM) (Fig. 1D), suggesting a mutation-selective therapeutic window. In Ba/F3 models, potency was markedly increased under IL-3–independent conditions, with > 30-fold shifts observed in triple-mutant EGFR cells (Fig. 1E, F) and double-mutant EGFR cells (Supplementary Fig. 1E and F), further underscoring its dependence on oncogenic EGFR signaling. By contrast, other targeted agents, including brigatinib [23], produced only an approximately 4-fold shift in the same system (Fig. 1G), and conventional chemotherapeutics showed little pathway selectivity (Fig. 1H, I). Osimertinib also showed increased potency under IL-3-independent conditions, with a substantial fold shift in Ba/F3 EGFR-LT cells (Fig. 1J). Collectively, these findings establish abemaciclib as a functionally reprogrammed inhibitor with pronounced selectivity for mutant EGFR signaling, highlighting its potential to overcome resistance-associated EGFR alterations.

**Fig. 2:**
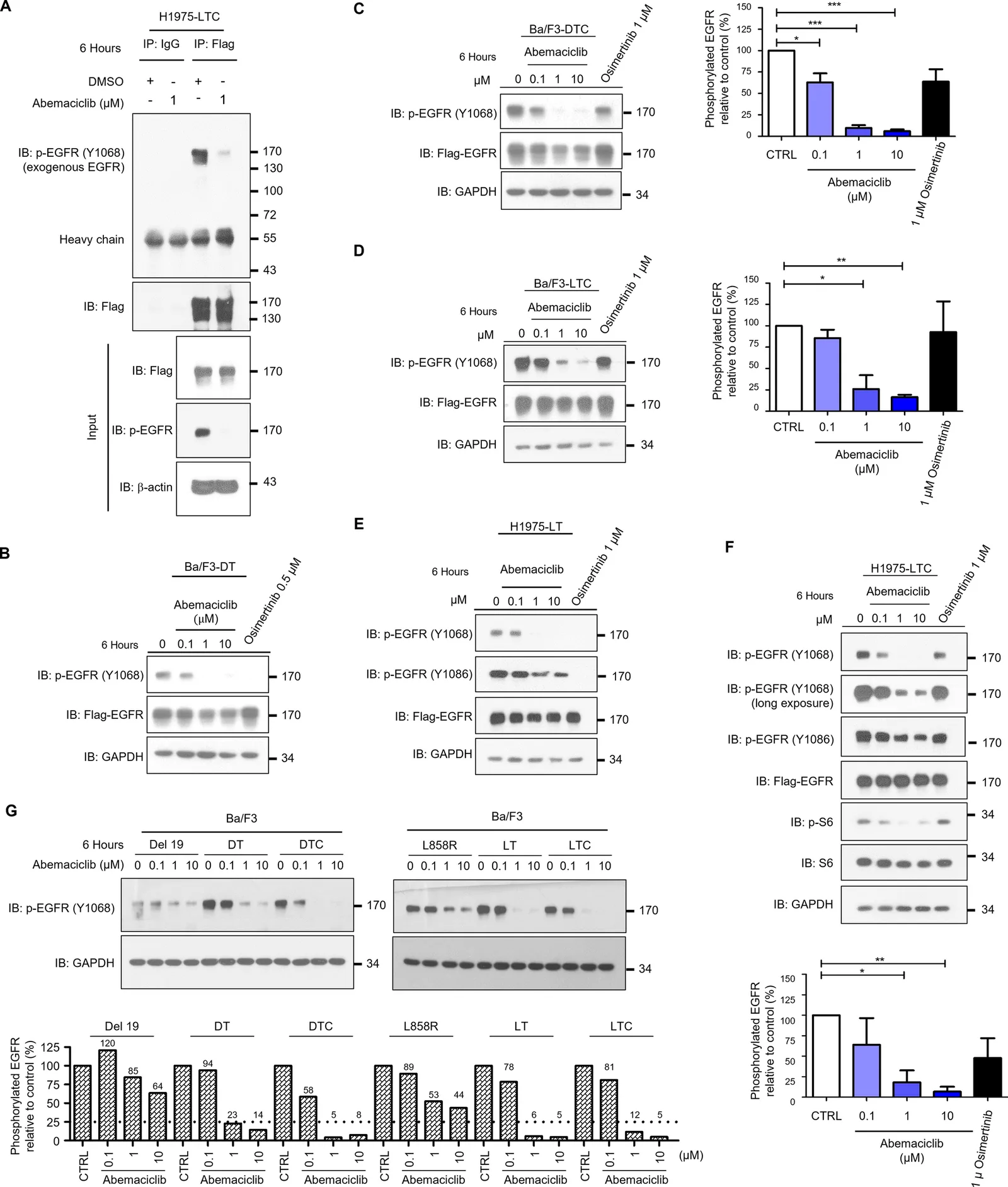
Abemaciclib suppresses phosphorylation of osimertinib-resistant EGFR triple mutants in vitro. Effect of abemaciclib on phosphorylated EGFR levels. **A** Immunoprecipitation (IP) and Western blotting were performed in H1975-LTC cells treated with abemaciclib for 6 hours, and the levels of phosphorylated EGFR were analyzed. β-actin served as a loading control. Effects of abemaciclib or osimertinib on phosphorylated EGFR levels. After 6 hours of abemaciclib incubation, phosphorylated EGFR levels were determined in Ba/F3-DT (**B**), Ba/F3-DTC (**C**), Ba/F3-LTC (**D**), H1975-LT (**E**), H1975-LTC (**F**) cells and phosphorylated S6 levels were determined in H1975-LTC (**F**) by Western blotting. Quantitative plots are shown on the right (**C** and **D**) or below (**F**) and each experiment was performed in three independent replicates, and the values for each treatment group were normalized to the corresponding control within the same experiment. Each treatment group was compared with the control using a two-tailed one-sample Student‘s t-test. Multiple comparisons were accounted for using the Benjamini-Hochberg (BH) false discovery rate (FDR) correction and an adjusted P value<0.05 was considered statistically significant. **G** The levels of phosphorylated EGFR were examined in BaF3-Del 19, BaF3-DT, BaF3-DTC, BaF3-L858R, BaF3-LT, BaF3-LTC cells by Western blotting, and quantitative plots are shown below. The error bars represent the means ± SDs; *n*=3

Consistent with its growth-suppressive activity, abemaciclib increased the sub-G1 population in both H1975-LTC and H1975-LT cells, accompanied by the cleavage PARP [30], indicating apoptotic cell death (Fig. 1K–M and Supplementary Fig. 1G). To directly assess its effect on EGFR activity, exogenous EGFR was immunoprecipitated from H1975-LTC cells and analyzed for phospho-EGFR (Y1068). Abemaciclib treatment markedly reduced EGFR phosphorylation, supporting its direct suppression of receptor activation (Fig. 2A). This inhibitory effect was observed across distinct EGFR mutational contexts, as abemaciclib consistently suppressed autophosphorylation of both double- and triple-mutant EGFR in Ba/F3 and lung cancer models in a dose-dependent manner (Fig. 2B–G). Concurrently, this treatment led to a dose-dependent suppression of the downstream EGFR signaling cascade, as evidenced by decreased levels of phosphorylated S6 (Fig. 2F). In contrast, osimertinib failed to effectively inhibit triple-mutant EGFR phosphorylation. Collectively, these findings demonstrate that abemaciclib directly attenuates mutant EGFR signaling and induces apoptotic cell death, supporting its repositioning as a mutation-selective EGFR inhibitor.

### Abemaciclib directly engages and selectively inhibits osimertinib-resistant mutant EGFR

**Fig. 3:**
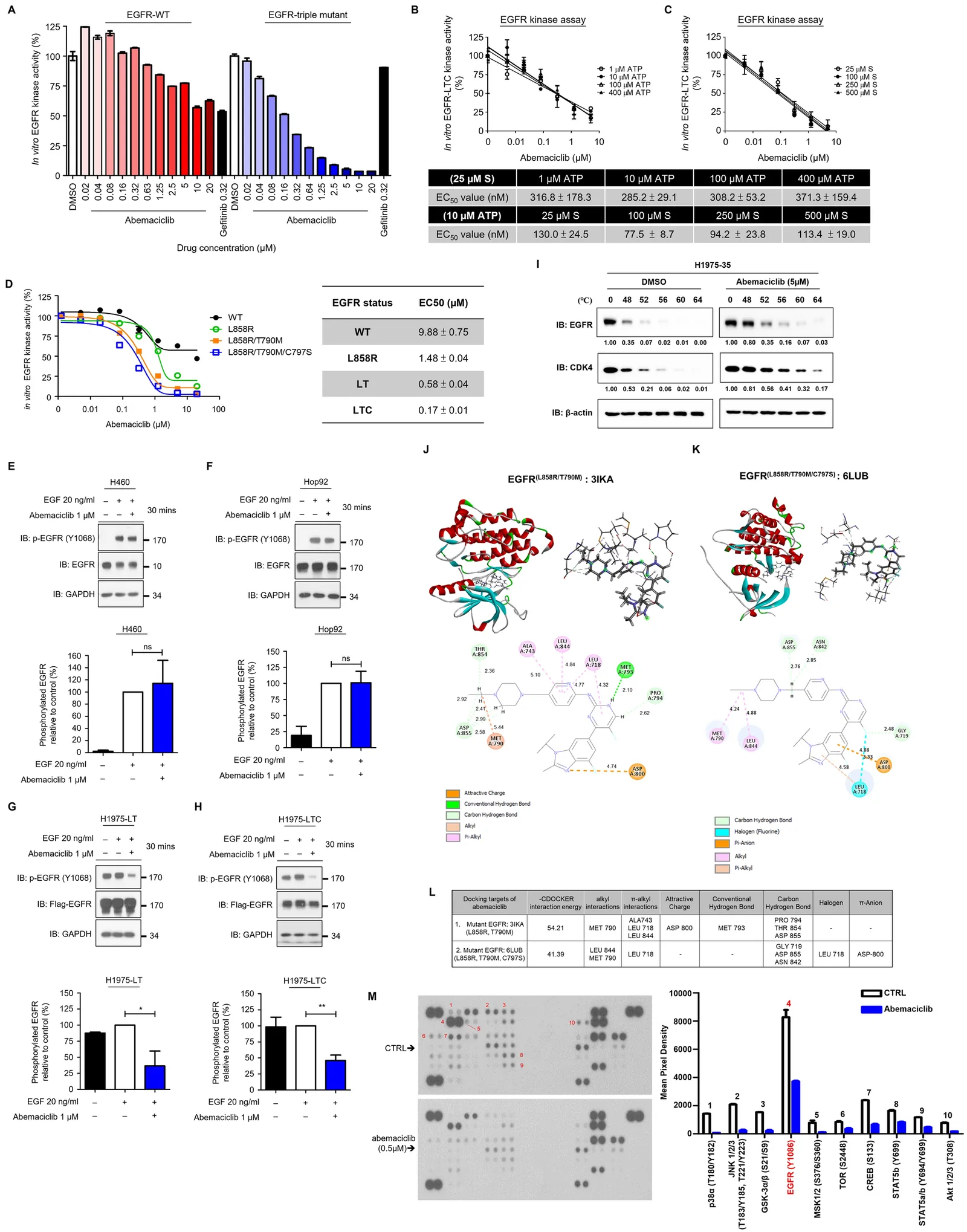
Abemaciclib selectively inhibits mutant EGFR and directly engages resistant EGFR variants. **A** An in vitro kinase assay was performed to measure the kinase activity of EGFR-WT and EGFR-LTC proteins treated with abemaciclib at the indicated concentrations. EGFR-LTC kinase activity was determined *in vitro* to measure the kinase activity of EGFR-LTC protein after treatment with abemaciclib, along with a relatively high concentration of ATP (**B**) or substrate (**C**). The IC50 values are presented below the chart. **D** An *in vitro* kinase assay was conducted to measure the kinase activity of the WT, L858R, LT and LTC-EGFR proteins treated with abemaciclib at the indicated concentrations. Effect of abemaciclib on phosphorylated EGFR levels in cells treated with or without EGF. The levels of phosphorylated EGFR were examined in H460 (**E**), Hop92 (**F**), H1975-LT (**G**) and H1975-LTC (**H**) cells by Western blotting. Quantitative plots are shown below. Each treatment group was compared with the EGF ligand stimulation group using a two-tailed one-sample Student's t-test. Multiple comparisons were accounted for using the Benjamini-Hochberg (BH) false discovery rate (FDR) correction and an adjusted P value < 0.05 was considered statistically significant. GAPDH served as a loading control in all the Western blotting panels. **I** A cellular thermal shift assay (CETSA) was performed in H1975-35 cells. H1975-35 cells were treated with DMSO or abemaciclib and subjected to the indicated temperature gradient (0–64°C). Cell lysates were analyzed by Western blotting for EGFR, CDK4, and β-actin. Band intensities were quantified by densitometry and normalized to the 0 °C condition. β-actin was used as a loading control. The binding mode of abemaciclib complexed with the EGFR L858R/T790M (PDB ID: 3IKA) (**J**) and EGFR L858R/T790M/C797S (PDB ID: 6LUB) structures (**K**) was analyzed by molecular docking. A summary table is shown (**L**). **M** Phosphokinase array analysis (#ARY003B; R&D Systems) was performed after treatment with abemaciclib. The bar graph showed differences of more than 2-fold between the abemaciclib-treated and control groups

To define the molecular basis of the mutation-selective activity of abemaciclib, we first performed *in vitro* kinase assays using purified EGFR proteins. Abemaciclib potently inhibited the kinase activity of triple-mutant EGFR, whereas its effect on wild-type EGFR was substantially weaker (Fig. 3A), indicating a pronounced preference for mutant conformations. Notably, the inhibitory potency was largely unaffected by increasing ATP or substrate concentrations (Fig. 3B, C), suggesting that abemaciclib does not act through canonical ATP competition but instead engages EGFR via a distinct mechanism. This mutation-selective inhibition was further confirmed in cellular systems. Abemaciclib failed to suppress EGFR activation in wild-type EGFR-expressing lung cancer cells, even under ligand stimulation (Fig. 3D), whereas robust inhibition was observed in double- and triple-mutant EGFR models (Fig. 3E–H). In contrast, other CDK4/6 inhibitors, including palbociclib [31] and LEE011 [32], did not show comparable suppression of EGFR signaling (Supplementary Fig. 2 A and B), leaving the levels of phosphorylated EGFR and phosphorylated AKT unaffected. Nonetheless, these inhibitors retained their canonical activity, as demonstrated by the reduction of phosphorylated Rb (Supplementary Fig. 2B) and decreased cell viability within mutant EGFR systems (Supplementary Fig. 2 C and D). Collectively, these results highlight a unique functional property of abemaciclib.

To directly assess target engagement in cells, we next performed cellular thermal shift assays (CETSAs). Abemaciclib markedly increased the thermal stability of EGFR at elevated temperatures, consistent with its direct binding in cells (Fig. 3I). A similar stabilization profile was observed for CDK4, in line with its known target engagement. Molecular docking further revealed that abemaciclib forms multiple stabilizing interactions with double- and triple-mutant EGFR, including hydrogen bonding, hydrophobic contacts, and Pi-interactions within the kinase domain (Fig. 3J–L). Abemaciclib exhibits binding behavior distinct from classical ATP-competitive EGFR inhibitors. Moreover, a human phosphokinase array identified EGFR phosphorylated at Y1086 as one of the principal targets of abemaciclib (Fig. 3M and Supplementary Table 1). This finding was independently confirmed by Western blotting, which demonstrated a robust, dose-dependent reduction in EGFR-Y1086 phosphorylation following abemaciclib treatment (Fig. 2E, F). Consistent with these results, abemaciclib dose-dependently inhibited EGFR phosphorylation at both Y1068 and Y1086. This multi-site inhibition indicates that abemaciclib exerts a robust, comprehensive blockade on the EGFR kinase domain itself, thereby offsetting both growth (Y1068-mediated) and migratory (Y1086-mediated) [33] signaling cascades in mutant EGFR systems. Together, these data demonstrate that abemaciclib directly engages mutant EGFR through a distinct binding mechanism, providing structural and functional support for its selective inhibition of resistance-associated EGFR variants.

### Abemaciclib suppresses tumor growth in xenograft models harboring EGFR triple mutations

**Fig. 4:**
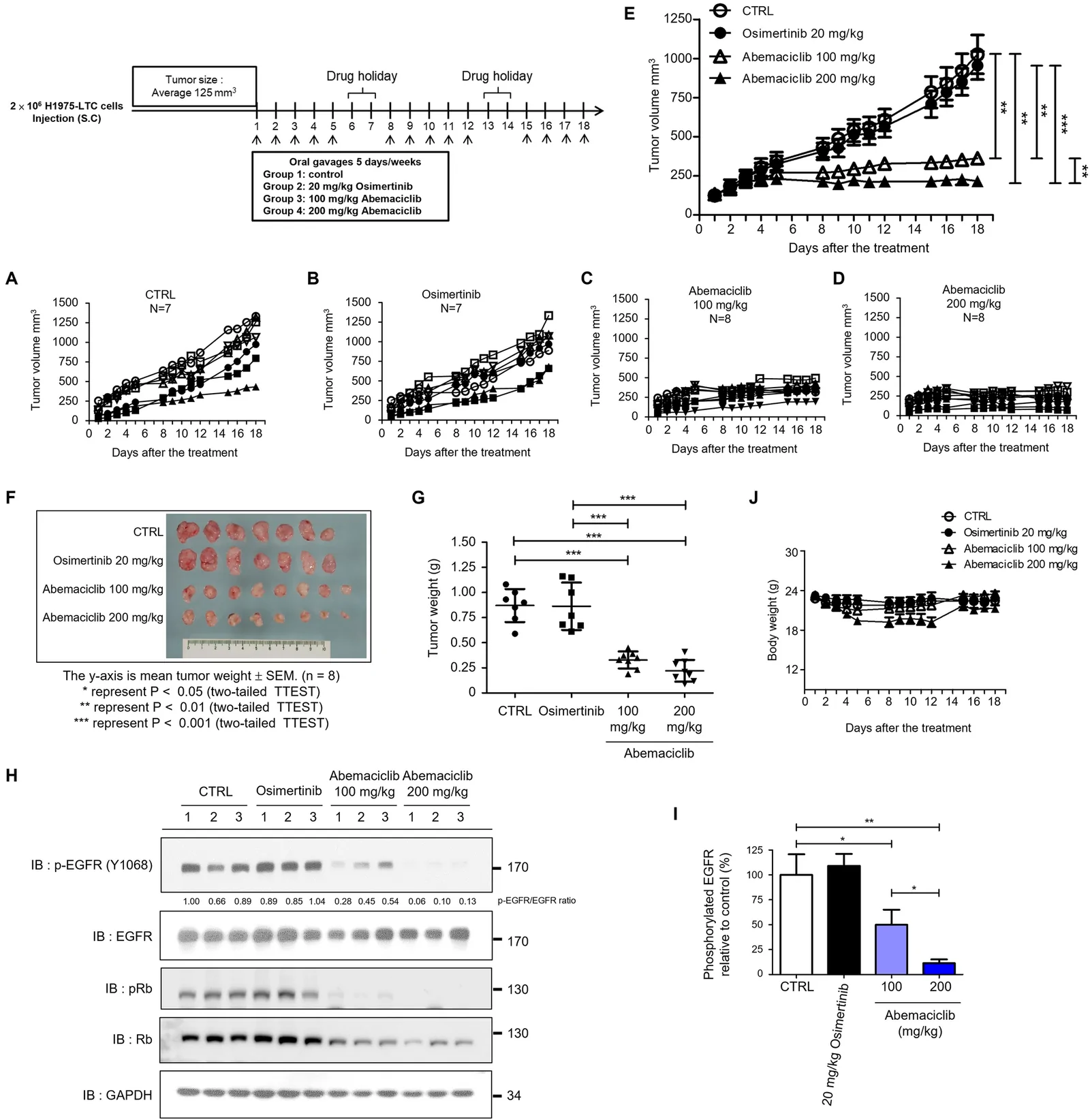
Abemaciclib suppresses tumor growth in xenograft models harboring EGFR L858R/T790M/C797S mutations. A schematic representation of the experimental procedure of the animal model is shown at the top. (**A**–**E**) Effect of oral gavage of abemaciclib or osimertinib on tumor growth. The tumor volume was measured as described in the Materials and Methods (n = 6 mice per group). (**F–G**) After the in vivo assay was performed, the mice were sacrificed, and the net tumor weights were measured. The data are presented as the means ± SEMs. * *P* < 0.05, ** *P*< 0.01, and *** *P* < 0.001. **H** Abemaciclib decreased p-EGFR levels in H1975-LTC xenograft tumors. The protein extracts from the tumor sections were subjected to Western blotting. **I** Effects of abemaciclib or osimertinib oral gavage on mouse body weight. **J** Abemaciclib decreased p-EGFR levels in H1975-EGFR-LTC xenograft tumors. The protein extracts from the tumor sections were subjected to Western blotting

To determine whether the mutant-selective activity of abemaciclib observed in vitro translated into therapeutic efficacy in vivo, we established xenograft models using H1975 cells stably expressing triple-mutant EGFR (L858R/T790M/C797S; EGFR-LTC) (Fig. 4). After subcutaneous implantation, mice bearing established tumors (~125 mm^3^) were treated orally with abemaciclib or osimertinib according to the indicated schedule. Abemaciclib induced a pronounced and dose-dependent suppression of tumor growth, with both 100 and 200 mg/kg significantly reducing tumor volume and final tumor weight relative to vehicle controls (Fig. 4A–G). In contrast, osimertinib failed to inhibit EGFR-LTC tumor growth, consistent with the refractory phenotype conferred by the C797S resistance mutation. Notably, osimertinib retained antitumor activity in double-mutant EGFR-LT xenografts (Supplementary Fig. 3), confirming that resistance in the LTC model was specifically associated with acquisition of the third-site mutation.

Mechanistically, tumors harvested from abemaciclib-treated mice showed marked suppression of EGFR phosphorylation and reduction of Rb phosphorylation. In contrast, osimertinib exerted minimal inhibitory activity against triple-mutant EGFR signaling in vivo (Fig. 4H, I). Importantly, despite its sustained antitumor efficacy, abemaciclib did not induce detectable systemic toxicity, as indicated by stable body weight and unchanged serum ALT, AST, BUN, and CRE levels throughout treatment (Fig. 4J and Supplementary Fig. 4A-H). Collectively, these findings demonstrate that abemaciclib suppresses C797S-mediated osimertinib resistance and exerts potent in vivo antitumor activity through suppression of mutant EGFR signaling in triple-mutant EGFR-driven tumors.

### Abemaciclib inhibits endogenous EGFR C797S signaling and induces apoptosis in CRISPR-engineered isogenic models

To exclude potential artifacts associated with ectopic EGFR overexpression and establish a genetically faithful resistance platform, we generated CRISPR/Cas9-edited isogenic H1975 and HCC827 cell lines harboring endogenous triple-mutant EGFR alleles, including H1975-35, H1975-12 (EGFR-LTC), and HCC827-2 (EGFR-DTC) cells (Supplementary Fig. 5 A). EGFR dependency of these engineered models was confirmed by shRNA-mediated EGFR depletion, which markedly reduced EGFR signaling and cell viability (Supplementary Fig. 5B and C), supporting continued oncogenic addiction to mutant EGFR despite acquisition of osimertinib resistance.

**Fig. 5:**
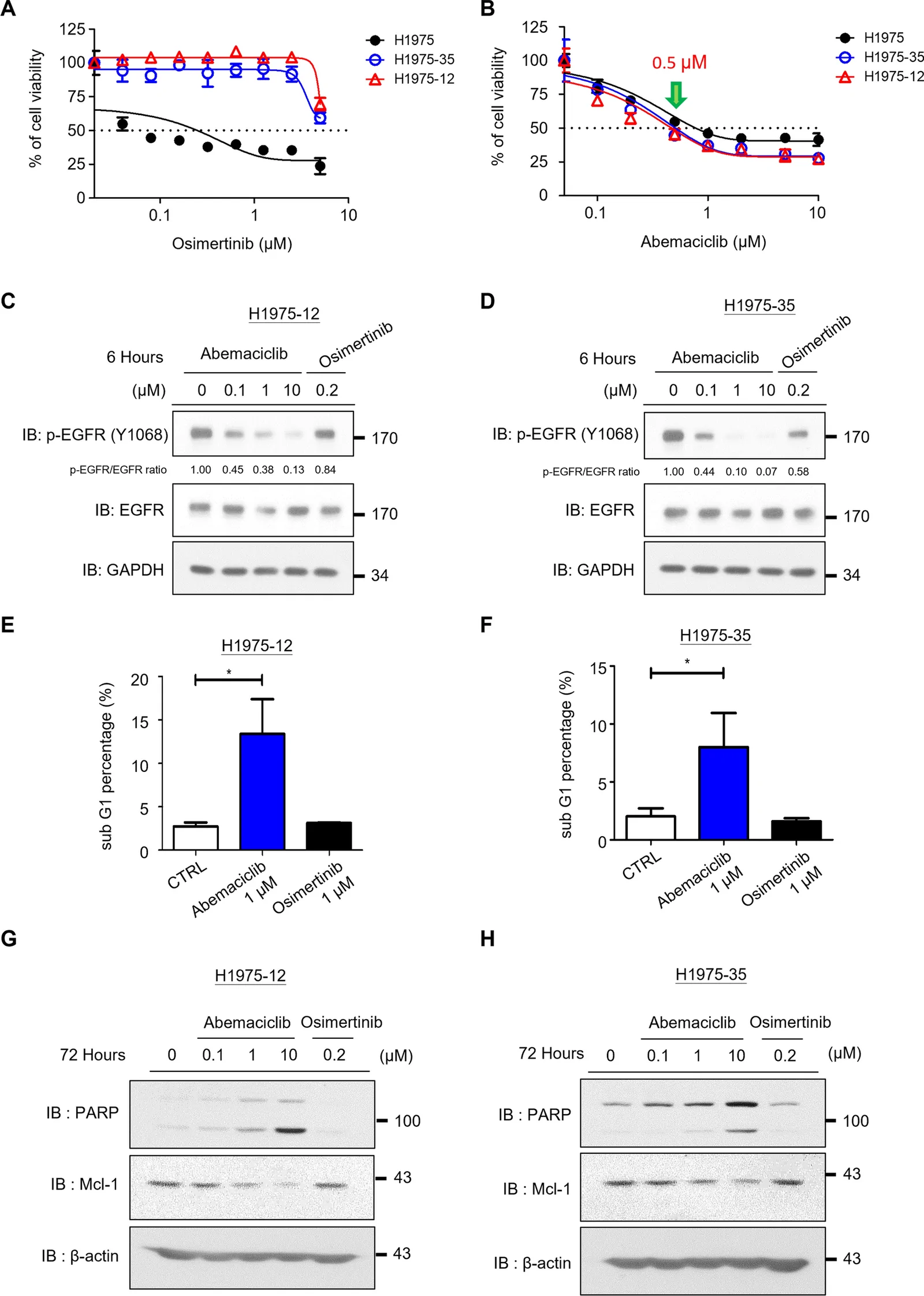
Abemaciclib inhibits mutant EGFR signaling and induces apoptosis in CRISPR-engineered EGFR C797S isogenic models. H1975 and isogenic cells (H1975-12 and H1975-35) were treated with osimertinib (**A**) or abemaciclib (**B**) at the indicated concentrations for 72 hours, and cell viability was analyzed using the MTS assay. Effects of abemaciclib or osimertinib on phosphorylated EGFR levels. The levels of phosphorylated EGFR were examined in H1975-12 (**C**) and H1975-35 (**D**) cells by Western blotting and were quantified using ImageJ software. GAPDH served as a loading control. The proportions of H1975-12 (**E**) and H1975-35 (**F**) cells in the sub-G1 phase were determined after abemaciclib or osimertinib treatment. H1975-12 (**G**) and H1975-35 (**H**) cells were treated with the indicated concentrations of abemaciclib or osimertinib for 72 hours, after which the cell lysates were harvested. Protein extracts were immunoblotted with the indicated antibodies. β-Actin served as a loading control in all the Western blotting panels

Consistent with clinical resistance phenotypes, osimertinib exhibited minimal antiproliferative activity in isogenic EGFR-LTC cells (Fig. 5A and Supplementary Fig. 5D). In contrast, abemaciclib potently suppressed the viability of both parental and triple-mutant EGFR isogenic cells, with IC₅₀ values converging at approximately 0.5 µM (Fig. 5B). Mechanistically, abemaciclib induced a robust dose-dependent reduction in EGFR phosphorylation, whereas osimertinib failed to effectively suppress mutant EGFR signaling (Fig. 5C, D). This signaling inhibition was accompanied by marked apoptotic induction, including accumulation of sub-G1 populations, PARP cleavage, and a decrease in the antiapoptotic protein Mcl-1[34] (Fig. 5E–H), indicating that abemaciclib triggers apoptotic collapse in osimertinib-resistant EGFR-driven cells.

**Fig. 6:**
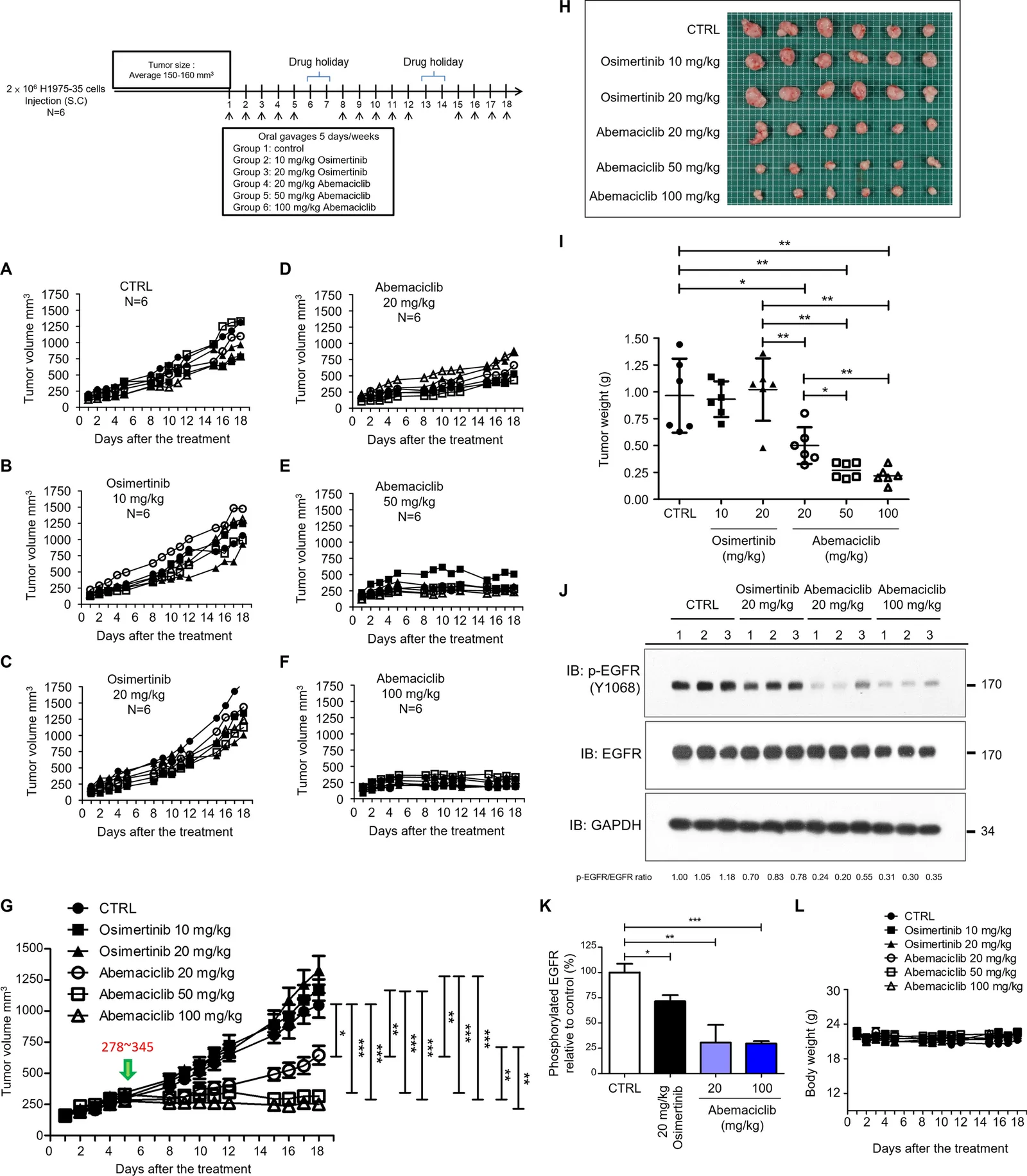
Abemaciclib demonstrates potent antitumor activity in EGFR C797S isogenic xenograft models. A schematic representation of the experimental procedure of the animal model is shown at the top. (**A**–**H**) Effect of oral gavage of abemaciclib or osimertinib on the tumor growth of the H1975 isogenic line. The tumor volume was measured as described in the Materials and Methods (n=6 mice per group). **I** After the in vivo assay was performed, the mice were sacrificed, and the net tumor weights were measured. The data are presented as the means ± SEMs. * *P* < 0.05, ** *P* < 0.01, and *** *P* < 0.001. (**J**–**K**) Abemaciclib decreased p-EGFR levels in H1975-LTC isogenic xenograft tumors. The protein extracts from the tumor sections were subjected to Western blotting. **L** Effects of oral gavage of abemaciclib and osimertinib on mouse body weight

We next evaluated the therapeutic efficacy of abemaciclib in vivo using H1975-35 xenograft tumors harboring endogenous EGFR-LTC mutations. Daily oral administration of abemaciclib at 20, 50, or 100 mg/kg produced substantial tumor suppression across all treatment groups, whereas osimertinib failed to control tumor growth (Fig. 6A–I). Importantly, abemaciclib-treated tumors displayed markedly reduced EGFR phosphorylation in vivo, further confirming direct pathway inhibition within resistant tumors (Fig. 6J, K). No overt systemic toxicity was observed, as body weight remained stable and serum liver/kidney function markers were unchanged during treatment (Fig. 6L and Supplementary Fig. 6A-J). Similar mutant-selective inhibitory effects were also observed in HCC827-2 cells harboring Del19/T790M/C797S EGFR mutations, in which abemaciclib suppressed EGFR phosphorylation and effectively inhibited xenograft tumor growth in vivo (Supplementary Fig. 7A-D). Together, these data establish that abemaciclib retains potent antitumor activity across genetically engineered endogenous EGFR triple-mutant models and is effective against both L858R- and Del19-based resistant EGFR backgrounds.

### Abemaciclib demonstrates therapeutic efficacy in patient-derived models of EGFR C797S-mediated osimertinib resistance

**Fig. 7:**
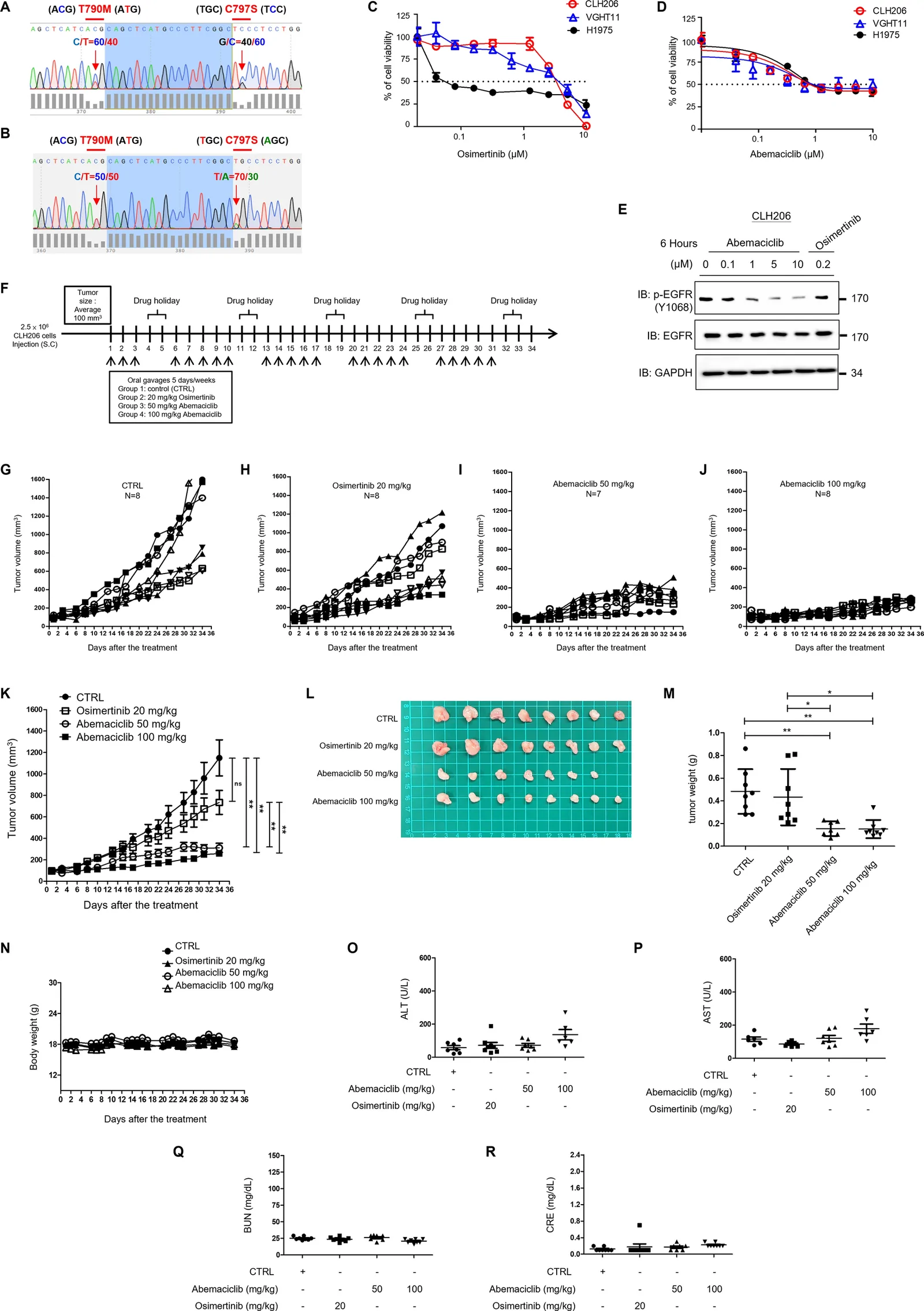
Abemaciclib inhibits tumor growth in a PDX model harboring EGFR-DTC mutations both in vitro and in vivo. Sequencing analysis of CLH206 cells, which express the EGFR Del19/T790M (+/**−**: 40/60)/C797S (+/−: 60/40) mutant (**A**) or VGHT11 cells, which express the EGFR Del19/T790M (+/**−**: 50/50)/C797S (+/**−**: 30/70) mutant (**B**). CLH206 cells and VGHT11 were treated with osimertinib (**C**) or abemaciclib (**D**) at the indicated concentrations for 72 hours, and cell viability was analyzed using the MTS assay. **E** Effects of abemaciclib or osimertinib on phosphorylated EGFR levels in CLH206 cells, as determined by Western blotting. GAPDH served as a loading control. **F** A schematic representation of the experimental procedure of the animal model. (**G**–**K**) Effect of oral gavage of abemaciclib or osimertinib on the growth of CLH206 tumors. The tumor volume was measured as described in the Materials and Methods (n = 8 mice per group). (**L**–**M**) After the in vivo assay was performed, the mice were sacrificed, and the net tumor weights were measured. The data are presented as the means ± SEMs. * *P* < 0.05, ** *P* < 0.01, and *** *P* < 0.001. **N** Effects of oral gavage of abemaciclib or osimertinib on the body weights of the mice. The levels of ALT (**O**), AST (**P**), BUN (**Q**), and CRE (**R**) in CLH206 tumor-bearing mice treated with abemaciclib or osimertinib, excluding those in mice with hemolyzed blood, were evaluated

To further assess the translational relevance of our findings, we evaluated abemaciclib in a patient-derived osimertinib-resistant triple-mutant EGFR (DTC) xenograft tumor model of CLH206 (Fig. 7A) or VGHT11 cells (Fig. 7B) isolated from patient-derived lung tumors harboring clinically acquired EGFR alterations. Consistent with its resistant phenotype, osimertinib exhibited limited inhibitory activity against CLH206 cells and VGHT11 cells, whereas abemaciclib robustly suppressed cell proliferation (Fig. 7C, D). Mechanistically, abemaciclib induced dose-dependent inhibition of EGFR phosphorylation in CLH206 cells, while osimertinib failed to effectively suppress mutant EGFR signaling (Fig. 7E), further supporting the mutant-selective activity of abemaciclib across clinically relevant resistant contexts.

We next established CLH206 patient-derived xenograft tumors to evaluate therapeutic efficacy in vivo. Following establishment of tumors (~100 mm^3^), mice received daily oral treatment with abemaciclib or osimertinib. Strikingly, abemaciclib treatment at both 50 and 100 mg/kg significantly suppressed tumor growth and reduced tumor weight compared with those of the vehicle and osimertinib-treated groups (Fig. 7F–M). In contrast, osimertinib failed to control tumor progression, consistent with persistent resistance mediated by triple-mutant EGFR. Importantly, abemaciclib treatment was well tolerated, with no detectable changes in body weight or serum ALT, AST, BUN, and CRE levels during treatment (Fig. 7N–R), indicating an absence of overt systemic toxicity. Collectively, these findings demonstrate that abemaciclib exerts robust antitumor activity across overexpression-based, CRISPR-engineered isogenic, and patient-derived triple-mutant EGFR models, supporting its potential as a mutation-selective therapeutic strategy to overcome osimertinib resistance in EGFR-driven lung adenocarcinoma.

## Discussion

**Fig. 8:**
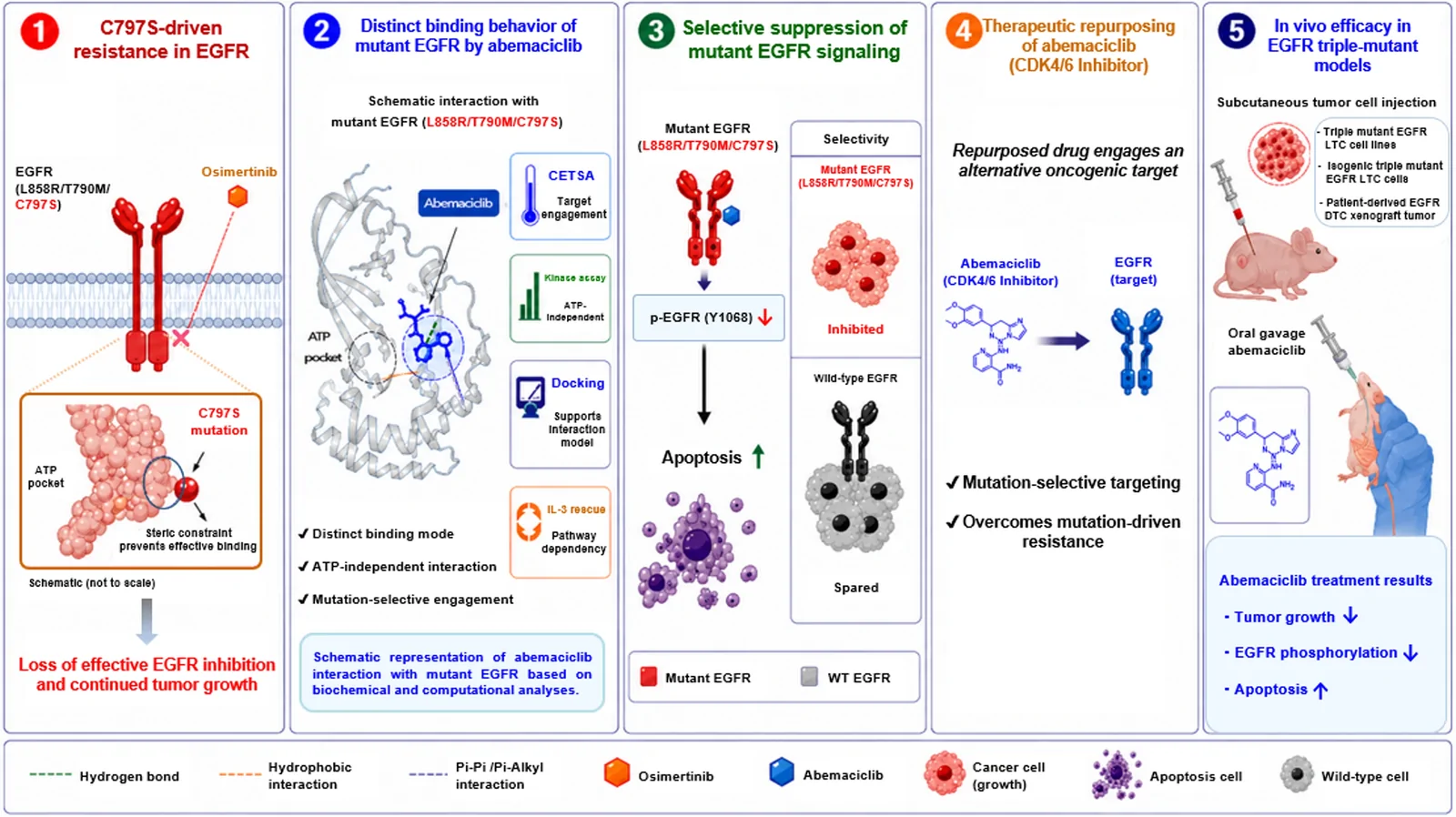
Proposed model of mutation-selective suppression of C797S-mutant EGFR by abemaciclib. Acquisition of the EGFR C797S mutation confers resistance to osimertinib and sustains oncogenic EGFR signaling. Through high-throughput screening, abemaciclib was identified as a potent suppressor of C797S-containing EGFR variants. Biochemical assays, CETSA analyses, and molecular docking studies support direct engagement of mutant EGFR by abemaciclib. Inhibition of mutant EGFR signaling results in reduced EGFR phosphorylation, induction of apoptotic cell death, and suppression of tumor growth in CRISPR-engineered and patient-derived xenograft models of osimertinib-resistant EGFR-mutant NSCLC. This schematic summarizes the proposed mechanism and therapeutic implications of abemaciclib in overcoming EGFR C797S-mediated osimertinib resistance. The model is based on experimental observations and does not represent an experimentally resolved binding structure

Our findings support a mechanistic model in which abemaciclib is functionally repurposed to selectively target mutant EGFR through a distinct binding mode of engagement. Unlike conventional EGFR inhibitors that rely on defined binding configurations and are susceptible to resistance-conferring mutations such as C797S, abemaciclib appears to exploit altered structural and conformational features present in mutant EGFR. The ATP-insensitive inhibition observed in biochemical assays, together with CETSA-based target engagement and docking-supported interaction patterns, suggests that abemaciclib interacts with EGFR in a manner distinct from classical ATP-competitive inhibitors. This interaction is preferentially manifested in mutant EGFR contexts, where structural perturbations induced by activating and resistance mutations may create or stabilize alternative binding interfaces. Such a mechanism provides a plausible explanation for the observed mutation-selective inhibition and the preserved activity against C797S-containing variants. Importantly, this model is further supported by the strong dependence of its cytotoxic effects on EGFR signaling, as demonstrated by IL-3 rescue assays, which link target engagement to functional pathway suppression. Notably, this property is not shared by other CDK4/6 inhibitors, indicating that the chemical scaffold of abemaciclib confers a unique capacity for dual-target engagement. Together, these observations define a strategy in which existing kinase inhibitors can be selectively suppressed toward resistant EGFR variants. This “therapeutic repurposing” mechanism expands the therapeutic strategy of drug repurposing and provides a generalizable strategy for overcoming mutation-driven resistance in kinase-targeted therapies. Leveraging its established safety profile as an FDA-approved drug [35], abemaciclib has emerged as a promising therapeutic for overcoming C797S-mediated osimertinib resistance (Fig. 8).

Brigatinib, an ALK inhibitor, is used to treat ALK-positive metastatic NSCLC after crizotinib progression but is less effective in patients with EGFR Del19 than in those with the L858R mutation [23]. Allosteric inhibitors such as EAI045 exhibit high WT EGFR selectivity and potency against L858R/T790M/C797S but are ineffective against Del19 variants [13, 24, 36]. Abemaciclib inhibits both the L858R/T790M/C797S and Del19/T790M/C797S mutations. No approved therapies have demonstrated consistent clinical efficacy against C797S-containing triple-mutant EGFR, and abemaciclib offers a promising alternative to overcome osimertinib resistance at C797S.

Abemaciclib (LY2835219), a selective CDK4/6 inhibitor, was initially evaluated in colorectal cancer and acute myeloid leukemia models and has recently received FDA approval for HR+/HER2- breast cancer [26, 27]. Preclinical studies have employed high doses of drug (20–100 mg/kg in our model; 75–300 mg/kg in others [31]), surpassing conventional TKI doses (~1 mg/kg), yet clinical adverse events remain tolerable, enabling chronic administration for sustained target inhibition [37]. Using in vivo xenografts (Figs. 4, 6 and 7), oral gavage of abemaciclib robustly suppressed EGFR kinase activity, supporting dose reduction as a potential strategy in EGFR-dependent clonal malignancies. Pharmacokinetically, abemaciclib requires higher dosing than osimertinib (5–25 mg/kg) [4], mirroring elevated clinical regimens and distinct pharmacodynamic profiles between agents [38–40].

Abemaciclib permits continuous dosing owing to its favorable safety profile, which facilitates sustained target inhibition. Initial trials established the efficacy of monotherapy in advanced breast cancer, NSCLC, colorectal cancer, glioblastoma, and other solid tumors [31, 41–44]. In NSCLC models, compared with wild-type xenografts, KRAS-mutant xenografts exhibit heightened sensitivity, which aligns with the synthetic lethality after CDK4 ablation in KRAS-driven lung adenocarcinoma models [45]. In our study, EGFR mutations were identified as predictors of abemaciclib responsiveness. Furthermore, abemaciclib potentiates combination regimens with anticancer agents or radiotherapy [46–50]. Osimertinib resistance is typically polyclonal and driven by coexisting mechanisms, rationalizing combined cell cycle blockade with sustained inhibition of the EGFR pathway to delay or surmount resistance. In preclinical EGFR-mutant models, the CDK4/6 inhibitor abemaciclib restores sensitivity and suppresses proliferation via Rb pathway modulation; CDK4/6i + EGFR-TKI combinations yield additive/synergistic antitumor effects [51]. Ongoing trials are evaluating dual-targeting agents, e.g., osimertinib + abemaciclib (NCT04545710), and EGFR agents with cell cycle/MAPK inhibitors. Recent preclinical/translational data and phase II trials underscore the efficacy of abemaciclib in recurrent ER+ endometrial cancer [52].

Our study repurposed abemaciclib and confirmed its efficacy and specificity for EGFR-mutant cancers; further research may be essential to thoroughly elucidate the mechanism of action of abemaciclib. Since clinical trials and real-world applications have indicated the safety and efficacy of abemaciclib in the treatment of breast cancer and other malignancies [31, 43], its potential alternative use as a monotherapy or combination therapy to overcome osimertinib resistance in EGFR-mutant lung adenocarcinoma has been supported.

## Conclusions

The EGFR C797S mutation is a major mechanism of acquired resistance to osimertinib in EGFR-mutant non–small cell lung cancer. Despite extensive efforts to develop next-generation EGFR inhibitors, effective therapeutic options for C797S-mediated resistance remain limited, highlighting the need for clinically translatable treatment strategies. In the present study, we used high-throughput screening and multiple preclinical models to identify the FDA-approved CDK4/6 inhibitor abemaciclib as a selective inhibitor of C797S-mutant EGFR. Abemaciclib directly engages mutant EGFR, suppresses oncogenic signaling, and demonstrates robust antitumor activity in CRISPR-engineered and PDX models of osimertinib-resistant disease. These findings provide a readily translatable therapeutic repurposing strategy for EGFR C797S-mediated resistance and illustrate how approved targeted agents can be leveraged to overcome resistance-associated EGFR alternations.

## Supplementary Information

Supplementary Material 1

Supplementary Material 2

## Data Availability

No datasets were generated or analysed during the current study.

## Abbreviations

TKI: Tyrosine kinase inhibitor
LTC: L858R/T790M/C797S
DTC: Del19/T790M/C797S
WT: Wild-type
PDX: patient-derived xenograft
NSCLC: Non-small cell lung cancer
FDA: Food and drug administration
uHTS: Ultra High-Throughput Drug Screening
