# Supplementary material for: Repurposing of abemaciclib to overcome C797S-mediated osimertinib resistance in EGFR-mutant lung adenocarcinoma
Source: J Biomed Sci. 2026 Nov 12;33:96. doi: 10.1186/s12929-026-01297-0 (PMC13756994; doi:10.1186/s12929-026-01297-0)
Supplement: Supplementary file 1 [file 12929_2026_1297_MOESM1_ESM.docx]

Supplementary Figures: Supplementary Figures 1- 7

**Repurposing of Abemaciclib to Overcome C797S-Mediated Osimertinib Resistance in EGFR-Mutant Lung Adenocarcinoma**

*Author Names*

Hsin-Jung Li^1^, Chao-Chi Ho^2^, Ci-Ling Chen^3^, Chi-Yuan Chen^4,5^, Ting-Jen Rachel Cheng^6^, Ya-Hsuan Chang^7^, Yu-Han Huang^7^, Ming-Shiu Lin^1,2^, Zi-Ling Lin^3^, Shuenn-Chen Yang^8^, Shih-Chien Huang^1^, Yu-Ya Hung^8^, Kuo-Yen Huang^±1,3,9^* and Pan-Chyr Yang^±1,2,10^*

^±^These co-corresponding authors are equal contributors to this paper

**Supplementary Figure 1.** EGFR-TKI-resistant Ba/F3 cells stably expressing triple mutant EGFR were established. (A) Effect of Ba/F3 cells expressing different types of EGFR on phosphorylated EGFR levels. Protein extracts were immunoblotted with the indicated antibodies. GAPDH served as a loading control in all Western blotting panels. Cell viability was analyzed using an MTS assay in Ba/F3 cells expressing different types of EGFR treated with IRESSA (B) or osimertinib (C) at the indicated concentration for 72 hours. (D) An in vitro kinase assay was performed to measure the kinase activity of EGFR-LTC cells treated with different drugs from a library of ~125,000 compounds. Ba/F3 EGFR-LT cells (E) or EGFR-DT cells (F) were treated with abemaciclib in the absence or presence of IL-3 at the indicated concentration for 72 hours, and cell viability was analyzed using the MTS assay. (G) H1975-LT cells were treated with the indicated concentration of abemaciclib for 48 hours, after which the cell lysates were harvested. Protein extracts were immunoblotted with the indicated antibodies. β-Actin served as a loading control in all the Western blotting panels. WT: wild type; D: Del19; T: T790M; C: C797S; LR: L858R.

**Supplementary Figure 2.** Abemaciclib directly suppresses mutant EGFR. (A) An in vitro kinase assay was conducted to measure the kinase activity of the LTC-EGFR proteins treated with abemaciclib, palbociclib, or lEE011. The levels of phosphorylated EGFR were examined in Ba/F3 EGFR-LTC cells. (B) Effect of abemaciclib, palbociclib, or lEE011 on phosphorylated EGFR, phosphorylated Rb, and phosphorylated AKT levels in HCC827-2 cells by Western blotting. H1975-LTC (C) and isogenic cells (H1975-35) (D) were treated with abemaciclib, palbociclib, or lEE011 at the indicated concentrations for 72 hours, and cell viability was analyzed using the MTS assay.

**Supplementary Figure 3.** Osimertinib treatment increases the EFGR-LT capacity and reduces the tumor size in a xenograft model. (A) The viability of H1975 cells expressing different EGFR variants treated with osimertinib at the indicated concentration for 72 hours was analyzed via an MTS assay. (B) The effect of different types of EGFR expression on phosphorylated EGFR levels in H1975 cells. Protein extracts were immunoblotted with the indicated antibodies. GAPDH served as a loading control in all the Western blotting panels. (C) Effect of oral gavage of osimertinib on the tumor growth of the H1975 isogenic line. Tumor volume was measured as described in the Materials and Methods (n=6 for each group). (D and E) After the in vivo assay, mice were sacrificed, and tumor net weights were measured. The data are shown as the mean ± SEM.

**Supplementary Figure 4.** Antitumor growth effects of abemaciclib on triple-mutant EGFR-overexpressing H1975 cells. (A-D) Effect of oral gavage of abemaciclib or osimertinib on the body weight of mice. The levels of ALT (E), AST (F), BUN (G) and CRE (H) in H1975-LTC cells treated with abemaciclib or osimertinib were evaluated.

**Supplementary Figure 5.** Establishment of the H1975 C797S isogenic line and abemaciclib suppression of EGFR activity and induction of cancer cell death in this isogenic line. (A) Endogenous EGFR-C797S isogenic H1975 (H1975-35) cell lines were generated by CRISPR/Cas9 editing. (B) Phosphorylated EGFR, phosphorylated AKT or phosphorylated ERK levels were evaluated in EGFR knockdown cells by Western blotting. GAPDH served as a loading control. (C) Cell viability was analyzed via an MTS assay in EGFR-knockdown H1975 or H1975-35 cells. (D) The effect of abemaciclib or osimertinib on phosphorylated EGFR, phosphorylated AKT or phosphorylated ERK levels in H1975 or H1975-35 cells as determined by Western blotting. GAPDH served as a loading control.

**Supplementary Figure 6.** Antitumor growth effects of abemaciclib on triple-mutant EGFR-overexpressing H1975-35 cells. (A-F) Effect of oral gavage of abemaciclib or osimertinib on mouse body weight. The levels of ALT (G), AST (H), BUN (I) and CRE (J) in H1975-35 cells treated with abemaciclib or osimertinib were evaluated.

**Supplementary Figure 7.** Abemaciclib induces significant tumor suppression in the Del19/T790M/C797S-mutant EGFR isogenic line HCC827-2 in a mouse xenograft model. (A) Phosphorylated EGFR levels were measured in HCC827-2 cells by Western blotting, and quantitative plots are shown below. GAPDH served as a loading control. (B) Schematic representation of the experimental procedure of the animal model. (C) Effect of oral gavage of abemaciclib on the tumor growth of the HCC827-2 isogenic line. The tumor volume was measured as described in the Materials and Methods section (n=2 for each group). (D) Mice were sacrificed after the *in vivo* assay, and tumor weights were measured. The data are shown as the mean ± SEM. Protein extracts from tumor sections were subjected to Western blotting. GAPDH served as a loading control.
